# What Influences the Perceived Trust of a Voice-Enabled Smart Home System: An Empirical Study

**DOI:** 10.3390/s21062037

**Published:** 2021-03-13

**Authors:** Yuqi Liu, Yan Gan, Yao Song, Jing Liu

**Affiliations:** 1 School of Design, Kyushu University, Fukuoka 8158540, Japan; liu.yuqi.design@gmail.com; 2 School of Mechanical Science and Engineering, Huazhong University of Science and Technology, Wuhan 430074, China; 3 School of Design, The Hong Kong Polytechnic University, Hong Kong 999077, China; yao.song@connect.polyu.hk; 4 Department of Industrial Design, Faculty of Design and Architecture, Universiti Putra Malaysia, Selangor 43400, Malaysia; gs58135@student.upm.edu.my

**Keywords:** internet of things, smart home, smart speaker, perceived trust, empirical study, structural equation modeling

## Abstract

Contemporarily, almost all the global IT giants have aimed at the smart home industry and made an active strategic business layout. As the early-stage and entry-level product of the voice-enabled smart home industry, the smart speakers have been going through rapid development and rising fierce market competition globally in recent years. China, one of the most populous and largest markets in the world, has tremendous business potential in the smart home industry. The market sales of smart speakers in China have gone through rapid growth in the past three years. However, the market penetration rate of related smart home devices and equipment still stays extremely low and far from mass adoption. Moreover, the market sales of smart speakers have also entered a significant slowdown and adjustment period since 2020. Chinese consumers have moved from early impulsive consumption to a rational consumption phase about this early-stage smart home product. Trust in the marketing field is considered an indispensable component of all business transactions, which plays a crucial role in adopting new technologies. This study explores the influencing factors of Chinese users’ perceived trust in the voice-enabled smart home systems, uses structural equation modeling (SEM) to analyze the interaction mechanism between different variables, and establishes a perceived trust model through 475 valid samples. The model includes six variables: system quality, familiarity, subjective norm, technology optimism, perceived enjoyment, and perceived trust. The result shows that system quality is the essential influence factor that impacts all other variables and could significantly affect the perceived trust. Perceived enjoyment is the most direct influence variable affected by system quality, subjective norm, and technology optimism, and it positively affects the perceived trust in the end. The subjective norm is one of the most distinguishing variables for Chinese users, since China has a collectivist consumption culture. People always expect their behavior to meet social expectations and standards to avoid criticism and acquire social integration. Therefore, policy guidance, authoritative opinions, and people with important reference roles will significantly affect consumers’ perceived trust and purchase intention. Familiarity and technology optimism are important influential factors that will have an indirect impact on the perceived trust. The related results of this study can help designers, practitioners, and researchers of the smart home industry produce products and services with higher perceived trust to improve consumers’ adoption and acceptance so that the market penetration rate of related products and enterprises could be increased, and the maturity and development of the voice-enabled smart home industry could be promoted.

## 1. Introduction

Driven by the rapid development of information communication technology, a new upgrading era of the global home appliance industry has arrived [1]. As one of the most potential Internet of Things (IoT) industries, the smart homes will have a profound social effect and tremendous business potential in the coming future [2]. With the popularization of Artificial Intelligence (AI) and sonic interaction technology, smart speakers, the early-stage product and control hub of the voice-enabled smart home system, have gone through explosive growth in recent years [3]. According to data released by Strategy Analytics, global smart speakers shipments reached 147 million units in 2019, with an increased rate of 70% compared with 2018 [4]. In 2020, due to the worldwide COVID-19 pandemic, smart speakers’ shipment has been cut down but has still maintained growth. In the first quarter, the global sales of smart speakers reached 28.2 million units, with an increase of 5% [5]. This number came to 30 million units in the second quarter with a rise of 6%. This is mainly related to the lifting of the blockade in the Asia-Pacific region and economic growth recovery, especially China [6]. For the third quarter, the global shipment was 35.6 million, and it increased into 58.2 million in the fourth quarter [7,8]. The market share of global smart speakers is primarily concentrated in the six internet giants, namely Amazon, Google, Baidu, Alibaba, Xiaomi, and Apple. They occupy more than 80% of the global market [9]. Among them, Baidu, Alibaba, and Xiaomi are Chinese IT companies whose leading enterprise focuses on the domestic consumers. Their large share and prominent position in the global market indicates that China is one of the largest markets globally for voice-enabled smart home products and services [10].

According to the China Electronics Standardization Institute, the Chinese smart homes market is growing at a rate of 20–30% each year. There is still massive space for continuous development in the future [11]. According to All View Cloud (AVC), Chinese smart speakers market sales in 2019 were 36.822 million units with a yearly increase of 126.6%, and sales were 6.91 billion CNY with an annual increase of 89.3% [12]. It is believed that the reason for the high growth of smart speakers is that the domestic internet giants continue to provide high subsidies to their IoT business. The low price brought by price wars and the increased brand recognition through popular entertainment programs have strongly stimulated consumers’ desire to buy [12]. Before the outbreak of the epidemic, AVC predicted that with the reduction of price subsidies, the smart speaker industry will return to rationality in 2020 and maintain a high growth rate of about 30%. The epidemic has had a specific impact on smart speakers’ overall sales, and the growth rate has declined more than expected [12]. The International Data Corporate (IDC) revealed that the Chinese smart speaker market sold 36.76 million units in 2020 with a yearly decrease of 8.6% compared to 2019; among them, smart speakers with screen accounted for 35.5%, and its sales increased by 31.0% [13]. Although relevant agencies are optimistically predicting that the smart speaker market will continue to expand [14], compared with the booming period from 2015 to 2019, the whole market growth has entered a significant slowdown and adjustment phase. Different from the previous low-cost attraction and the freshness and enthusiasm for emerging technologies, Chinese consumers have become more rational currently. One crucial phenomenon that needs to be noted is that although the smart speaker is the early-stage product of the voice-enabled smart home system, the majority of consumers use it as a music player, while a few of them apply the voice assistant for home automation control [3]. This is because the whole smart home system is still in its realization period, technology stays immature, users’ consumption awareness has not been cultivated, functions and services remain monotonous, etc. If the smart home industry and its business ecosystem want to achieve further development, technological breakthroughs, experience optimization, content upgrading, functions and services diversity, etc., are necessary in the future.

In social science, trust is a kind of dependency relationship. Stakeholders involved in the connection (such as companies and customers) trust each other and rely on other parties to realize their desires and wishes [15]. In the marketing field, it is considered an indispensable component of all business transactions and regularly remains the research focus of new technology adoption [16]. This is especially true for transactions with uncertain conditions, since customers will face potentially unfavorable choices and risk costs. For the smart home industry, it is still in the early development stages and presents uncertainties regarding technology reliability and service quality. Users’ perceived trust plays an essential role in overcoming perceived risks and mitigating uncertainty for smart homes adoption. Therefore, consumers’ trustworthiness regarding the voice-enabled smart home system is the essential influencing factor for their attitude and intention to use [17,18,19]. For this paper, we define trust as the users’ confidence in the reliability of smart homes to meet their needs and expectations.

This study explores the influencing factors of Chinese users’ perceived trust in the voice-enabled smart home system, uses structural equation modeling (SEM) to analyze the interaction mechanism between different variables, and establishes a perceived trust model through 475 valid samples. The model mainly includes six variables, namely system quality, familiarity, subjective norm, technology optimism, perceived enjoyment, and perceived trust. The result shows that system quality is the essential influence factor that impacts all other variables and positively affects the perceived trust. Perceived enjoyment is the most direct influence factor affected by system quality, subjective norm, and technology optimism, and it positively affects the perceived trust in the end. The subjective norm is one of the most distinguishing variables for Chinese users, since China shares a collectivist consumption culture. Chinese people always expect their behavior to meet social expectations and standards to avoid criticism and acquire social integration. Therefore, policy guidance, authoritative opinions, and people with important reference roles will substantially affect consumers’ perceived trust and purchase intention. Familiarity and technology optimism are important influential factors that will have an indirect impact on the perceived trust. The related results of this study can help designers, practitioners, and researchers of the smart home industry come out with products and services with higher perceived trust. It can improve consumers’ adoption and acceptance of voice-enabled smart home systems so that the market penetration rate of related products and enterprises could be increased, and the maturity and development of the smart home industry could be promoted.

## 2. Literature Review

### 2.1. Development Status of Smart Home

#### 2.1.1. Development History of Voice-Enabled Smart Home System

Despite the optimistic expectation of future market growth, the smart home industry’s development status is still in its early and realization phase. The products and services are still far from mass-market phase adoption [3]. However, contrary to this fact, the market of global smart speakers has gone through explosive growth in recent years. The smart speaker, an early-stage product and control center of smart homes, is a wireless speaker and voice command device with an integrated virtual assistant. Users can request it to play music, broadcast the weather, set the alarm, control smart home devices, etc., through voice commands. Some smart speakers are also equipped with a touch screen that is called a smart display. The “fire” of smart speakers spread from Amazon. In November 2014, Amazon launched a new product, the smart speaker Echo, which integrates sonic interaction into the traditional loudspeaker and endows it with the attributes of artificial intelligence [20]. The voice assistant is called “Alexa”, which can play music, broadcast news, do online shopping, call Uber taxis, order takeaways, control home appliances, and communicate with users such as friends, etc. Subsequently, Google, Microsoft, and Apple entered the smart speaker industry, launched their relevant products, and became prominent global market players.

The situation is different in China, where concentration in the smart speaker industry is intensified by three domestic companies, Alibaba, Baidu, and Xiaomi. They stand out and occupy up to 95.6% of the whole market share, while international companies share shallow penetration [21]. This phenomenon is mainly due to the following reasons. First, Chinese enterprises have advantages in local contexts and understand consumers’ psychology, behaviors, and demand better. Second, domestic smart speaker products have far more competitive prices than international products when their prices convert into Chinese Yuan (CNY) according to the international exchange rate. Third, there is a language difference. China’s official language is Chinese, which is one of the most complex languages globally, and it makes the voice input and output utterly different from the English context, which is the main focus of the international Internet companies. This leads to the situation in which local products have more significant language advantages and performance in speech and semantic recognition. Since the smart home industry stays immature in China, we will discuss the market development phrase of its early-stage product, the smart speakers. It could be divided into four stages. The first one is the bud stage from 2015 to 2016; the second one is the accumulation stage from 2017 to 2018; the third one is the outbreak stage from 2018 to 2019; and the fourth one is the rational stage from 2020 to the present.

- The first stage: bud stage (2015–2016). Chinese IT companies identified business opportunities from Amazon. In July 2015, the first technology giant Linglong Technology was quickly established by Iflytek and Jingdong (JD) in the four months after the launch of Amazon Echo, focusing on the smart speaker market. With the dual support of Iflytek’s voice technology and the strength of the JD e-commerce platform, Linglong Technology released the first smart speaker Dingdong one month after its establishment, which kicked off the prelude to the development of the Chinese smart speaker market. Relevant data show that Dingdong speakers monopolized the Chinese smart speaker market with a 65% share in 2016. Although Dingdong speakers have taken the lead position in the Chinese market at that time, the whole industry has not received much attention. The market model was not transparent, and many Internet companies were still waiting or accumulating commercial foundations. Moreover, the cost and price of smart speaker were still relatively high at this period (the price of the first Dingdong smart speaker was 798 CNY).
- The second stage: accumulation stage (2017–2018). In 2017, Alibaba, Xiaomi, and Baidu successively launched their first smart speaker product. Alibaba’s Tmall Genie received various subsidies and applied preferential strategies to control the product price under 100 CNY and fought fierce price wars during the “Double Eleven” festival. The start of this price war also means that the Chinese smart speaker market has entered the second phase. Alibaba, Xiaomi, and Baidu have all stepped down, using the strategy of investing significant amounts of money and providing subsidies to despoil market share, create market demand, and form user groups quickly. In 2018, as the price war gradually became more normalized [22], Linglong Technology, which was overwhelmed, divided up due to internal conflicts and withdrew from the market sadly. So far, the Chinese smart speaker market has officially entered the era of three dominant players, Alibaba, Xiaomi, and Baidu.
- The third stage: outbreak stage (2018–2019). After forming the top three players pattern, Chinese smart speakers development entered the third stage. In this period, the overall market size has grown by leaps and bounds and even rushed into the global head market. According to IDC data statistics, in 2018, Chinese smart speaker annual shipments exceeded 20 million units with a yearly increase of 1051.8%, marking the outbreak of the Chinese smart speaker market [23]. The total shipments of the Chinese smart speaker market in 2019 were 45.89 million units with a yearly increase of 109.7% [24]. The market share of the three top players, Alibaba, Baidu, and Xiaomi, exceeds 90% [24]. Among them, Alibaba’s Tmall Genie ranked first with 15.61 million units shipment throughout the year, which is a yearly increase of 87.9%. Baidu Xiaodu shipped 14.9 million units throughout the year with an increase of 278.5% compared to last year. Xiaomi Xiaoai shipped 11.3 million units throughout the year, which is a yearly increase of 89.7% [24]. Moreover, the Chinese smart speaker market also presents a tripartite state with the basic pattern of differentiated development of other IT companies, such as JD, Huawei, Himalaya, etc. The year 2019 has witnessed the turning point of smart speakers becoming a standard accessory for Chinese families. However, the potential and capability have not been fully explored yet.
- The fourth stage: rational stage (2020–present). Although the shipment volume of the Chinese smart speaker market is growing rapidly, compared with the principal developed countries, the penetration rate remains low. This is mainly because Chinese users have not cultivated their habits yet and are not sufficiently sticky with related usage scenarios. In 2019, the US smart speaker penetration rate was the highest, reaching 26% [25]. The user number of smart speakers in China ranks first globally, with nearly 86 million, but the penetration rate of smart speakers is only 10%, which is much lower than the United States. Followed by the United Kingdom, the scale of smart speaker users and the penetration rate is 13 million people with a 22% penetration rate. For other countries, Germany and Canada are more than 15% [26]. This also means that China still has a large amount of “black land” waiting to be reclaimed. Affected by the COVID-19 epidemic, although the market still maintains a high growth level, the growth trend has slowed down compared to 2019. In meeting the diversified needs of consumers, Alibaba, Baidu, and Xiaomi have added screen speakers, which have been an important driving force for developing the smart speaker market in the past two years and will become the next main battle field. The screen speaker has completed the leap from voice extension to vision, from single-mode to multi-mode interaction, especially when the product is superimposed with functions such as video, entertainment, learning, call, etc. Overall, the explosive market growth occupied in the early developing stage of the smart speaker industry is slowly disappearing. The market is gradually tending to a rational state while excellent system quality and service experience have gradually become a rigid demand for the expansion of the future market.

#### 2.1.2. Development Strategies of Chinese IT Giants for Smart Home

The division of the above four stages is only for the single product of smart speakers. When it comes to smart homes, the current market and industry are still in their initial development status, and the opening of the smart speaker market is only an early prologue for voice-enabled smart homes. Currently, the three Chinese IT giants, Alibaba, Baidu, and Xiaomi, are actively deploying their smart homes AIoT (AI + IoT) ecosystem via their advantages in smart speakers’ market share. In early 2020, Alibaba upgraded the Tmall Genie business to an independent business department. The Tmall Genie department held a 2020 spring conference and proposed the “Double Hundred Plan” in May. They announced that Alibaba would invest tens of billions to build content, services, and the AIoT ecosystem and further consolidate the growth soil of smart speakers. They will create hundreds of smart home devices to accelerate the explosion of the smart industry with the integration of the Alibaba Cloud’s IoT resources. Relying on Internet thinking and design, supply chain, brand, and channel sales capabilities, Xiaomi has accumulated a large quantity of user base, numerous equipment, massive data, and application scenarios to build its own AIoT ecosystem. At the beginning of the 20th century, the CEO of Xiaomi, Lei Jun, stated that Xiaomi’s investment in AIoT would be increased from 10 billion yuan to fifty billion yuan in five years. At the same time, he also determined the strategy of “5G + AI + IoT”. As the main entry product of the AIoT ecosystem, smart speakers are also valued by Xiaomi. Baidu has the world’s leading ABC (Artificial Intelligence, Big Data, Cloud Computing) and IoT technology. The guarantee of hard power allows Baidu to quickly gain the right of speech in the smart speaker industry and enter the first echelon. In 2019, Baidu focused on the screen speaker layout. With the excellent technical foundation, high-quality hardware products, and rich content resources, it quickly grabbed half of the screen speakers and maintains its leading role until now. By 2020, Baidu successively released three screen speakers, continuing to consolidate its leading position. Although the current Chinese smart speaker market is mainly occupied by three local Chinese Internet giants, the industrial ecosystem of the voice-enabled smart home is still in an early development stage. International Internet companies still have the opportunity to use their advantages to enter the Chinese smart home industry via different forms; for example, Microsoft could provide official work support, and Apple’s huge mobile share in the Chinese market is also its advantage in developing the smart home ecological chain.

### 2.2. Research on Trust

Trust is a multidisciplinary concept that scholars have given different definitions from different perspectives: sociology, psychology, organizational behavior, marketing, etc. In management, trust is usually considered to be related to the significant qualities of the trader’s integrity, honesty, and kindness, which help the determination and performance of contracts. It can reduce uncertainties and opportunistic behaviors in the transaction process, avoid legal procedures, minimize the costs of different parties, and promote the benefits of all stakeholders. In marketing, trust is mainly concentrated on relationship and channel management. Anderson and Narus argue that trust is a belief in which one party believes the other parties will perform behaviors that are beneficial to them without unexpected harmfulness of the trade relationship [27]. Trust has been considered a catalyst for buyer–sellers transactions that could reduce uncertainty and vulnerability in the exchange process [28,29,30,31]. Furthermore, trust has been shown to be a strong predictor of task performance for consumers using technology [32,33]. The establishment of trust is the key to promoting users’ purchase behavior and increasing satisfaction and brand loyalty. With regard to the acceptance of emerging technologies, trust occupies an important and essential status. Perceived trust toward a kind of new technology can be defined as the extent to which one believes the technology usage will be reliable and credible [34]. Compared with traditional products, purchasing emerging technologies products will make consumers face more technical and service uncertainties, instability, and potential hazards related to security and privacy. Due to the uncertain environment that consumers may face, perceived trust in relevant merchants and brands is crucial. It will significantly affects customers’ use and purchase intentions [35], which is especially typical for the smart home industry. Research shows that there is a positive relationship between residents’ perceived trust and attitude to use toward smart home services [18]. In recent years, research on consumers’ perceived trust in emerging technology has aroused wide interests in academia and has been applied in different technology fields, such as e-commerce [36], online retailing [37,38], internet banking [39], and mobile payments [40].

### 2.3. Theories and Models of Technology Adoption

With regard to technology adoption, there are a number of models and theories. In 1967, the Theory of Reasoned Action (TRA), a general theory of behavior prediction, was first introduced by Fishbein and Icek Ajzen [41]. They believe that behavioral beliefs determine people’s attitudes, normative beliefs determine subjective norms, attitudes and subjective norms determine behavioral intention and ultimately affect actual behavior. In 1985, Icek Ajzen found that people have control beliefs in the process of decision making. He improved the theory of TRA by adding a perceived behavioral control element and proposed the Theory of Planned Behavior (TPB) [42]. He believes that people’s decision-making includes consideration of behavioral risks and whether the perceived risk is under control stays an essential factor affecting people’s behavioral intention and actual behavior. In TPB, he introduced the concept of “perceived” and pointed out the subjectivity of people’s decision-making behavior. In 1989, Davis applied TRA and TPB to the field of information systems to explain users’ acceptance of information technology and proposed the Technology Acceptance Model (TAM) [43]. TAM regards system design features as external variables for users to decide whether to use the information system. External variables directly determine the perceived ease of use. Then, these two variables determine the perceived usefulness. The perceived usefulness and perceived ease of use determine people’s attitude toward using, which in turn affects people’s intention to use, and the intention to use ultimately determines the actual system use. The Motivation Model (MM) was used by Davis et al. to study the adoption of Information Communication Technology (ICT) in 1992 [44]. It believes that individual behavior is based on intrinsic and extrinsic motivation. In this model, computer playfulness and enjoyment are the determinants of intrinsic motivation. Perceived usefulness, perceived ease of use, and subjective norms are determinants of extrinsic motivation. The Innovation Diffusion Theory (IDT) was proposed by the American scholar Everett M. Rogers in 1962 about persuading people to accept new ideas, new things, and new products through the media. It focuses on new technologies and social behaviors to reveal the spread process of new technologies in society [45]. Social Cognitive Theory (SCT) started as Social Learning Theory (SLT) in the 1960s and developed into SCT in 1986 by Albert Bandura. The theory believes that learning takes place in a social environment, where personal factors, environmental factors, and behaviors have dynamic and mutual interactions [46]. It assumes that users acquire and maintain behaviors while considering the social environment of their developing behaviors and highlights the concept of self-efficacy. Thompson et al. proposed the Model of Personal Computer Utilization (MPCU) in 1991 to predict users’ personal computer usage behavior [47]. They suppose that users’ behavior depends on several aspects, their wishes (attitudes), what they think they should do (social norms), what they have usually done (habits), and the expected consequences of their behaviors. They identified six determinants of technology acceptance in the model, namely job fit, complexity, long-term consequences, affect toward use, social factors, and the facilitating conditions.

### 2.4. Research on Trust and Technology Adoption of Smart Home

Researchers have also integrated trust with different technology adoption theories and models. Yang et al. have developed a comprehensive model via extending the Theory of Planned Behavior (TPB). The model validates that mobility, security, privacy risk, and trust in the service provider are important factors affecting customers’ behavioral intentions to adopt and use smart home services [48]. Mulcahy et al. demonstrate a new predictive model of smart home technology adoption by bridging three important frameworks: the technology readiness index (TRI) 2.0, consumer engagement, and perceived risk and trust [49]. Shuhaiber extends the Technology Acceptance Model (TAM) and incorporates more factors related to users’ smart home adoption via PLS-SEM, an analysis method of Structural Equation Model (SEM) based on Partial Least Squares (PLS). The research validates that trust, awareness, enjoyment, and perceived risks, perceived usefulness, and perceived ease of use significantly influence customers’ attitude and intention to use smart homes [50]. Pal et al. develop and test a theoretical framework empirically to identify the core factors that affect the older adults’ acceptance of smart home services for healthcare and verify that trust has a significantly positive impact on the users’ behavioral intention [51]. Ferraris et al. define a trust model and propose a solution based on data collection and invasion of user privacy in smart home devices [52]. Cannizzaro et al. conduct a nationally representative survey to measure the adoption and acceptability of UK consumers about smart homes and underlined how businesses and policy makers should work together to improve the socio-technical affordances and strengthen consumers’ trust [53]. Kim et al. propose a trustworthy gateway system architecture that builds an Internet of Things trust domain to protect the smart home from malicious attack [11]. There are still many other types of research.

In general, for smart homes, the literature concerning trust is primarily analyzed from user adoption and acceptability, during which perceived trust is regarded as a single variable in the model framework. Few models specifically focus on users’ perceived trust analysis. Moreover, most of the researchers that focus on trust start from the perspective of engineering and computer science, which are mainly related to the privacy and security system development; few start from the perspective of consumers’ perception and decision-making. At present, there is a lack of research on the perceived trust of smart home systems, no need to mention taking the Chinese consumers as the targeted audience. Since China has gradually become one of the most prosperous smart home markets in the world, it is of high significance to explore the consumer decision-making and perceived trust factors of the Chinese group. This research fills up this gap.

## 3. Hypotheses Development and Research Framework

Following the theoretical review and the analysis of the development status of the voice-enabled smart home industry in China, we selected six variables as our research object to construct the perceived trust model of Chinese customers: system quality (SQ), familiarity (FAM), subjective norm (SN), perceived enjoyment (PE), technology optimism (TO), and perceived trust (PT). Due to the deduction based on existing facts and research studies, 14 hypotheses have been proposed, and the research framework has been formulated.

### 3.1. System Quality

For the products and services of voice-enabled smart homes, system quality is the essential and most important variable among all the factors influencing consumer perceived trust and decision-making behavior. The accurate identification of user commands, the diversity of system functions, the timely responsiveness of service, the user privacy and security protection, etc., are all determined by the system quality [54]. Excellent system quality should maximally respond to the diverse needs of users and reduce the perceived risks. For familiarity, a high-quality smart home system should take the users’ existing experience and recognition into consideration and improve the ease of use of products and services. For subjective norm, excellent system quality is an invisible business advertisement for smart homes to win policy support and public opinion promotion, which will indirectly reflect on invisible customers’ perceptions. For technology optimism, high-quality smart home systems will significantly enhance consumers’ optimistic attitude toward related technology due to its rich functions and excellent user experience. For the perceived enjoyment, outstanding system quality can improve the user’s efficiency, safety, and experience to enhance their sense of pleasure. For perceived trust, good system quality invisibly conveys a sense of reliability and remains the source of users’ perceived trust. Based on the above analysis, we put forward the following hypotheses:

**Hypothesis** **1** **(H1).** System quality positively affects the subjective norm.

**Hypothesis** **2** **(H2).** System quality positively affects the perceived trust.

**Hypothesis** **3** **(H3).** System quality positively affects the perceived enjoyment.

**Hypothesis** **4** **(H4).** System quality positively affects the technology optimism.

**Hypothesis** **5** **(H5).** System quality positively affects the familiarity.

### 3.2. Familiarity

Alba and Hutchison defined familiarity as the number of product-related/service-related experiences accumulated by the consumer, including direct and indirect experiences such as advertising exposures, interactions with salespersons, word of mouth communications, trial and consumption [55]. Following this definition, smart home system familiarity is defined as the accumulated related experiences and recognition customers have had with smart homes. Studies have shown that familiarity influences the consumers’ decision-making process [56,57], the relationship between price and quality [58], and advertising effectiveness [59]. Fundamentally speaking, users prefers to accept things and experiences they are familiar with, while strange things are more resistant. For subjective norm, familiarity will act on the cognition of subjective norm on consumers. For perceived enjoyment, things with higher familiarity are closer to the users’ comfort zone, which can help users reduce the necessity of changes and gain a higher sense of security and pleasure. For technology optimism, a deeper understanding of the possibilities and prospects of a particular technology could help the consumers gain recognition and optimistic attitude more easily. For perceived trust, familiar things are more trustworthy than strange things [60]. According to the discussion above, we come up with the following hypotheses:

**Hypothesis** **6** **(H6).** Familiarity positively affects the subjective norm.

**Hypothesis** **7** **(H7).** Familiarity positively affects the perceived enjoyment.

**Hypothesis** **8** **(H8).** Familiarity positively affects the perceived trust.

**Hypothesis** **9** **(H9).** Familiarity positively affects the technology optimism.

### 3.3. Subjective Norm

Subjective norm refers to the social pressure that a person feels about whether to take a particular behavior. It represents, when predicting people’s behavior, the influence that reference individuals or groups have on their decision-making [61]. Ajzen and Fishbein’s research shows that perceived stress and important reference groups, such as spouses or family members, can substantially impact consumers’ specific purchase behaviors [61]. Hee stated that such perceived stress is particularly significant in the context of Chinese culture [62]. Lam agreed with this and pointed out that in China, the code of behavior reinforces the avoidance of criticism and the desire to gain recognition through social integration [63]. Therefore, the subjective norm is crucial in explaining the wishes and behaviors of Chinese consumers. Since China’s traditional culture is a typical collectivist culture, Chinese consumers are primarily influenced by social policies, public opinion, and other important reference people when making purchase decisions. For perceived enjoyment, consumption and purchase behaviors that meet social expectations or being supported by important reference groups can give consumers a higher sense of pleasure. For perceived trust, it is easy for products and services supported by relatives or friends and recognized by public authorities to gain consumers’ trust. Taking the above analysis into consideration, we give the following hypotheses:

**Hypothesis** **10** **(H10).** Subjective norm positively affects the perceived trust.

**Hypothesis** **11** **(H11).** Subjective norm positively affects the perceived enjoyment.

### 3.4. Perceived Enjoyment

The concept of perceived enjoyment comes from hedonistic theory, which believes that perceived enjoyment is the ultimate goal of all behavior, and the purpose of personal action is to obtain pleasant emotions and avoid unpleasant or painful emotions [64]. Consumer research often describes hedonic emotions as the pleasure of consuming or using a product or service. Sometimes, this kind of consumption or use refers to behaviors related to multiple senses, imagination, and emotions during the product or service experience [64]. Menon and Kahn believe that perceived enjoyment is a positive psychological state, which refers to how a person feels pleasure, happy, joyful, or satisfied with a specific situation [65]. Marketing scholars and information system adoption researchers believe that perceived enjoyment is the internal motivation of personal emotions and plays a vital role in consumption experience [66]. In information technology research, perceived enjoyment refers to the degree to which an individual perceives pleasure and joy in the activity process while using a relevant computer system [43,67]. According to previous research, when personal behavior is driven by internal motivation such as interest and enjoyment, they are more willing to persist in the future [67]. For smart home systems, the ease of use of the system, the support of public opinion and government policies, and personal technology optimism and familiarity will all affect consumers’ perceived enjoyment and intention to use. Park et al. have validated that enjoyment was positively related to users’ adoption of smart homes via investigating the core motivations with the original technology acceptance model [19]. A similar result also has been acquired by Shuhaiber [50]. Users are more likely to form a sense of trust in products and services that have a high pleasure degree. Thus, we acquire the following hypotheses:

**Hypothesis** **12** **(H12).** Perceived enjoyment positively affects the perceived trust.

### 3.5. Technology Optimism

Technology optimism was formed at the end of the 19th century when some central European countries successively completed the industrial revolution, and the social function of technology became increasingly apparent. After the Second World War, the world’s new technology revolution has achieved significant progress. The Western economy has gained unprecedented prosperity. Plenty of sociologists began to accept technology optimism, which believes that the advance of technology development can determine the historical destiny of humanity. It can provide the material means for humanity to solve social problems. In this study, the technology optimism variable is derived from technology optimism theory, but it is defined as the users’ positive and optimistic attitude toward the social value and future development of the voice-enabled smart home industry. Technology optimism could be obviously affected by consumers’ characteristics, such as experience, education level, knowledge, recognition, etc., determining its strong subjective attributes. People who are optimistic about smart home technology are more likely to have a sense of pleasure in the use of related products and services, and this optimistic attitude will enhance users’ attitude to use and purchasing intention of related products and services. Therefore, we made the following hypothesis:

**Hypothesis** **13** **(H13).** Technology optimism positively affects the perceived enjoyment.

**Hypothesis** **14** **(H14).** Technology optimism positively affects the perceived trust.

**Figure 1:**
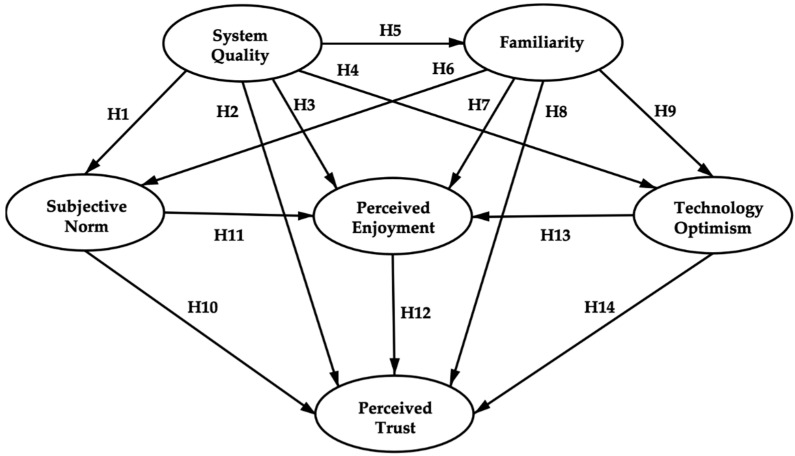
The conceptual framework of hypotheses.

According to the description and analysis above, the fourteen hypotheses and research framework have been developed, as shown in Figure 1. We argue that system quality, familiarity, subjective norm, perceived enjoyment, and technology optimism are the critical influencing variables of perceived trust. Their interaction mechanism can be shown as follows.

## 4. Methodology

To verify the model proposed in Figure 1, we conducted empirical research to explore the rationality of Hypotheses 1–14 and the interaction relationship between the six variables in the model.

### 4.1. Measurement Development

This research involves six variables: system quality, familiarity, subjective norms, technology optimism, perceived enjoyment, and perceived trust. The observed variables of all variables are all extracted from the previous study, and the specific content and related references are shown in Table 1. All the survey variables are required to measure their items on the 5-point Likert scale which is scored from 1–5, where “1” is “strongly disagree”, “2” is “disagree”, “3” is “neutral”, “4” is “agree”, and “5” is “strongly agree”.

### 4.2. Survey Procedure and Data Collection

After the measurement items were determined, we translated them into Chinese, added the demographic information questions, and developed a preliminary questionnaire. The questionnaire was randomly distributed through the Chinese Internet questionnaire platform, Wenjuanxing. Investigation and data collection are mainly divided into two stages, which include the pre-questionnaire test and the main study. In the first stage, we collected 50 initial questionnaire responses. Through the analysis of the data results, redundant items were deleted. Simultaneously, the question description language and overall logic were optimized to ensure that the respondents were more intuitive and accurate in obtaining the questions’ meaning and options set. Based on the adjustment of the pre-questionnaire test, we determined the final questionnaire content and started large-scale online delivery. The detailed content of the final questionnaire can be seen in Appendix A. In the end, a total of 664 formal questionnaires were collected, and users with relevant experience in using intelligent voice assistants or relevant smart home devices were identified as targeted samples that met the research requirements. Under this standard, 475 valid questionnaires were obtained, with an effective rate of 71.5%, as data samples for further analysis.

### 4.3. Data Analysis Plans

For data analysis, the research was initially conducted the descriptive analysis, reliability, and validity test. Then, model fit was checked to satisfy the relevant criteria. Lastly, SEM was performed to check path analysis for hypothesis evaluations. SPSS 25.0 was mainly responsible for summarizing the demographical information and the preliminary statistics, while AMOS 25.0 was mainly used to conduct factor analysis and path analysis.

### 4.4. Demographic Information

In the demographic information part, the study uses descriptive statistical analysis to obtain statistics on the basis information of the valid samples, including gender, age, education background, monthly income, etc. Among the 475 samples, 194 are males, accounting for 40.8% of the total sample; 281 are females, accounting for 59.2%. The detailed results are shown in Table 2.

## 5. Results and Findings

The model analysis results mainly consist of three parts, and the first one is descriptive analysis, reliability, and validity; the second one is model fit, and the third part is hypothesis testing and path analysis. The following will discuss the relevant data and results in detail.

### 5.1. Descriptive Analysis, Reliability, and Validity

Descriptive analysis was summarized in Table 3. For the reliability tests, Cronbach’s alphas served as an indicator for measuring the internal consistency of the survey with a threshold of 0.7 [83]. The adequate reliability could reveal that items reflect similar observations under similar scenarios with similar participants [84]. For validity tests, unidimensionality validity, convergent validity, and discriminant validity served as indicators for confirmative factor analysis [85], which measures the degree of association between a claimed factor and its operationalization, as depicted in Table 4. Results indicated that all 24 items had achieved sufficient validity. To specify, the threshold of standardized factor loadings and averaged variances expected (AVE) are both beyond 0.5 or above, and the composite reliability (C.R.) should be above 2 [86], indicating that the current sample has achieved satisfactory validity. In addition, maximum shared variance (MSV) and average shared variance (ASV) were examined to check whether discriminant validity is adequate. The relevant results are shown in Table 4.

### 5.2. Model Fit

Regarding the model fit, the goodness-of-fit (GFI) severed as an indicator by the following indices [87], namely standardized root mean square residual (SRMR), goodness-of-fit index (GFI), root mean square error of approximation (RMSEA), normed fit index (NFI), incremental fit index (IFI), Tucker–Lewis index (TLI), and comparative fit index (CFI). All the indices have achieved sufficient fitness within the thresholds, as shown in Table 5.

### 5.3. Hypothesis Testing and Path Analysis

**Figure 2:**
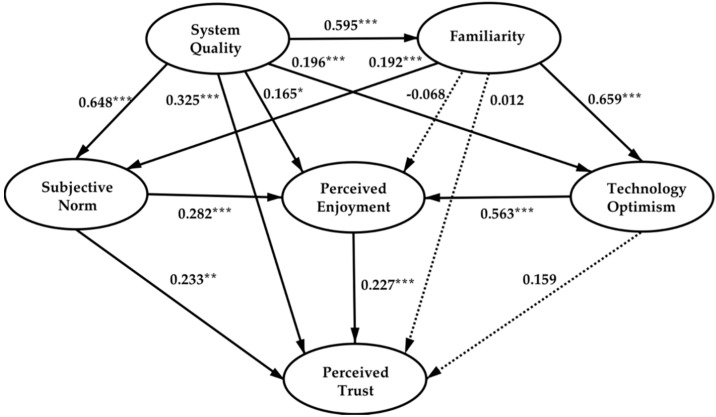
Path coefficients resulting from structural equation modeling (SEM). Note: * *p* < 0.1; ** *p* < 0.05; *** *p* < 0.01.

As to test Hypotheses 1–14, a path analysis was further conducted via structural equation model (SEM) to evaluate the association among various factors [88]. SEM in this study is a systematic approach to examine research hypotheses holistically [88]. Figure 2 and Table 6 show the results of path analysis and the examination of hypotheses 1–14.

## 6. Discussion and Implications

From the results of data analysis, it can be seen that system quality is the foundation for users to build perceived trust in voice-enabled smart home products and services. It has a significantly positive effect on all the model variables. System quality can significantly affect subjective norm, which means that excellent system quality is undoubtedly an invisible business card for products and services promotion. It can influence government policies support, public opinion, product reputation, praise, and the favorability of people who have close relationships or set typical references for the consumers to exert substantial influence on their perceived trust. Concerning perceived enjoyment, good system quality could be reflected in the interaction process between users and products. It can provide friendly operation experience, meet users’ value demand, invoke users’ pleasure emotions, such as good usability, reliability, compatibility, accuracy, timeliness, ease of use, etc. Moreover, system quality has a significantly positive influence on the technology optimism. A high-quality system could promote the favorability of relevant technology so that users are more likely to believe that related products can meet their demands and are more willing to use them. In addition, system quality has a significant impact on familiarity, which shows that good system quality is based on the familiar operation and cognition of users so that the user’s operation difficulty of products and services could be significantly reduced. System quality has a significant impact on perceived trust, which shows that good products and services can speak for themselves, the excellent quality could be felt during the user experience process, and the sense of trust emerged spontaneously. Related to the discussion above, Hypotheses H1–H5 were verified. It can be seen that although the Chinese smart speaker industry has experienced rapid growth in the past few years due to subsidies and cheap prices, the winners who can steadily occupy the Chinese smart home market are those who provide customers with high system quality and an excellent user experience.

Familiarity has a significant effect on subjective norm and technology optimism but has no direct effect on perceived enjoyment and perceived trust. This may be due to the immaturity of the current voice-enabled smart home technology and market, and the experience of early adopters with related products and services will feel distrustful because of the gap between the real product experience and their expectations. However, it may also be because the influence of familiarity on perceived trust in this model is distributed by other two variables, subjective norm and technology optimism. Here, H6 and H9 are verified; H7 and H8 are rejected. The voice-enabled smart home industry is currently in the early stage with technology instability, service uncertainty, and market immaturity. The users’ behavior and habits have not been fully cultivated. Therefore, the development of related products and service systems should start from the existing needs, cognition, and operating habits of consumers, embed new technology functions step by step, and thus cultivate user behavior habits and needs gradually to pave the way for the maturity of the market.

Subjective norm has a positive effect on perceived trust and will further affect perceived trust by its significant influence on perceived enjoyment. This is because the consumption behaviors of Chinese consumers are often affected by public opinion, product and service provider reputation, the advice and suggestions of important reference persons, as well as the government policy and authoritative viewpoints when making consumption decisions. Positive feedback from these people could psychologically help customers get rid of worries and greatly enhance their perceived trust and pleasure emotions; so Hypotheses H10 and H11 have been verified. With regard to subjective norm, companies can mainly make breakthroughs in marketing methods. On one hand, they can cooperate with governments, communities, enterprises, and institutions to order products in a mass quantity. On the other hand, they can seek diversified marketing and promotion methods, such as television advertisement, online shopping, social media, live broadcast platforms, etc. For example, currently, Chinese live broadcasts remain popular and hot; many live broadcast platforms have tremendous fans and audience bases in which many customers will follow the consumption guideline of individual broadcasting stars, etc. These methods can significantly affect potential consumers’ social and public opinion environment, thereby affecting their perceived trust in corresponding products and services.

Technology optimism has a significant effect on perceived enjoyment but has no direct effect on perceived trust. This is probably because technology optimism presents consumers’ positive attitude toward the overall development trend of voice-enabled smart homes in the future. This positive attitude is conducive to the generation of pleasurable emotions for products and services equipped with related technology. However, it does not directly reflect the perceived trust in current products, since there is a time gap of the innovation diffusion. Another possibility is that the utility of the technology optimism variable in this model for perceived trust has been completely transmitted through perceived enjoyment, which makes it exerts an indirect effect. That is why the direct path is not significant. So H13 was verified, and H14 was rejected.

Perceived enjoyment is directly affected by system quality, subjective norm, and technology optimism, and it significantly impacts perceived trust. This shows that among all the factors, the shaping of perceived enjoyment during the experience, usage, and purchase of a voice-enabled smart home system has the largest role in establishing perceived trust. H12 has been verified.

Through the discussion above, we can gain the implications for the voice-enabled smart home system. The system quality exerts a fundamental and essential effect on perceived trust. For the voice-enabled smart home industry, user-centric product function upgrade, technology reliability, compatibility development, service experience optimization, market standard-setting, etc., are eternal themes for the maturity of the smart home industry. Perceived enjoyment is the most direct and core variable that could be influenced by all the other variables and act on perceived trust in the end. Subjective norm plays a typically distinguishing role in establishing the trustworthy sense of Chinese consumers toward smart home devices. Familiarity and technology optimism are important factors that have indirect effects on perceived trust.

## 7. Limitations and Future Studies

This study mainly explored the perceived trust model of the voice-enabled smart home system of Chinese consumers, which has not examined the influence of individual characteristics factors on perceived trust. In the future, the influence of personality traits on the perceived trust of the smart home will be further discussed, which may include customer gender, age, education level, occupation, monthly income, family status, technology experience, etc. Accordingly, user types will be analyzed. Moreover, in this study, the hypotheses that familiarity and technology optimism could significantly affect perceived trust have not been verified. However, both of them directly or indirectly affect perceived enjoyment in this model, and perceived enjoyment has a significantly positive impact on perceived trust. Therefore, there is a high possibility that perceived enjoyment may play a mediation role between familiarity and perceived trust as well as technology optimism and perceived trust. In future study, we would like to further explore this assumption.

## 8. Conclusions

The world is under a new wave of technological and industrial revolution. The smart home industry represented by IoT smart devices has become the infrastructure of future smart cities and is an essential driving force of economic and social transformation. In recent years, the early-stage product and control hub of the voice-enabled smart home system, the smart speaker, has been undergoing rapid development, and the number of shipments has experienced unprecedented explosive growth. As a system with intelligent sonic interaction, Internet service content, the ability of countless devices expansion and services access, the voice-enabled smart home can significantly promote consumers’ well-being and improve their quality of life in the future. However, despite the high expectation, the market penetration rate of smart homes is still far from mass adoption. As the most populous country in the world, China has tremendous potential in the voice-enabled smart home industry, which could be seen from the local market performance of smart speakers over these years. The establishment of perceived trust is the basis for users’ adoption and purchase intention of emerging technology products and services. This study establishes a perceived trust model of Chinese consumers through path analysis and structural equation modeling based on the responses of 475 users who have relevant experience in using voice-enabled smart home devices. There are six variables in the model, namely system quality, familiarity, subjective norm, perceived enjoyment, technology optimism, and perceived trust. Among them, system quality is the essential and fundamental variable that affects perceived trust. Perceived enjoyment is the most direct and intuitive influencing variable, which could be directly or indirectly affected by all other variables. Subjective norm is the most representative influencing variable for the perceived trust of Chinese customers. Since Chinese culture is a kind of collectivist culture, people will always suppose that their behavior meets the expectations of the general public and society standards. Users’ familiarity with products and services and their optimistic attitude toward technology are important factors that directly and indirectly affect perceived trust. The results of this study provide important empirical evidence for the design, development, and research of the voice-enabled smart home system, which makes the study of high theoretical and practical value.

## Figures and Tables

**Table 1:** Constructs and measurement items.

Construct	Measure Item	Reference
System Quality (SQ)	SQ1: Voice-enabled smart homes enable me to control many applications and devices.	[19,54,68,69]
	SQ2: Voice-enabled smart homes perform their functions quickly, efficiently, and precisely.	
	SQ3: Voice-enabled smart homes and the output of information fully meet my needs.	
	SQ4: Voice-enabled smart homes can protect my privacy and security.	
Familiarity (FAM)	FAM1: I am familiar with the concept and related knowledge about voice-enabled smart homes devices.	[70,71,72]
	FAM2: I am familiar with the brands, products, and services of voice-enabled smart homes devices.	
	FAM3: I am familiar with how to operate voice-enabled smart homes devices.	
Subjective Norm (SN)	SN1: I will use voice-enabled smart homes devices if the media/government encourages me to use them.	[73,74,75]
	SN2: I will use voice-enabled smart homes devices in my house if my family members and friends do so.	
	SN3: I will use voice-enabled smart homes devices if people whose opinion I value recommend that I do so.	
	SN4: I will use voice-enabled smart homes devices if people who influence my behavior recommend that I do so.	
	SN5: I will use voice-enabled smart homes devices if people who are most important to me support me doing so.	
Perceived Enjoyment (PE)	ENJ1: Using voice-enabled smart homes would be fun.	[19,50,76,77,78]
	ENJ2: Using voice-enabled smart homes would be pleasurable.	
	ENJ3: Using voice-enabled smart homes would be enjoyable.	
	ENJ4: Using voice-enabled smart homes would make me excited.	
Technology Optimism (TO)	TO1: I feel the newest technologies contribute to a better quality of life.	[49,79,80,81]
	TO2: I feel that products and services that use the newest technologies are much more convenient to use.	
	TO3: I feel confident that the newest technology-based systems will follow through with what I instruct them to do.	
	TO4: I feel the newest technologies can allow me to tailor things to fit my own needs.	
Perceived Trust (PT)	PT1: I consider voice-enabled smart homes to be trustworthy.	[50,82]
	PT2: I consider voice-enabled smart homes to be reliable.	
	PT3: I consider voice-enabled smart homes to be controllable.	
	PT4: I consider voice-enabled smart homes to be competent.	

**Table 2:** Demographic information and attitude for home motion sensing game.

Attributes	Value	Frequency	Attributes	Value	Frequency
Gender	Male	194	Income (RMB)	<1000	102
	Female	281		1000–3000	31
Age	20-	71		3000–5000	75
	21–30	242		5000–7000	92
	31–40	108		7000+	175
	41–50	40	Occupation	Students	138
	51+	14		Teachers	22
Education	Some colleges	142		Civil Servants	35
	Undergraduate	291		Workers	22
	Postgraduate	42		Others	258

**Table 3:** Reliability and unidimensionality.

Construct	Cronbach’s Alpha	Variable	Mean	Standard Deviation	Standardized Factor Loading	C.R. (t-Value)	SMC	AVE	Composite Reliability
System Quality (SQ)	0.846	SQ1 SQ2 SQ3 SQ4	3.81 3.97 3.69 3.83	0.900 0.779 0.932 0.887	0.751 0.765 0.748 0.796	- 17.908 17.004 16.851	0.564 0.586 0.559 0.633	0.587	0.850
Familiarity (FAM)	0.853	FAM1 FAM2 FAM3	4.25 4.20 4.26	0.747 0.761 0.742	0.773 0.832 0.831	- 18.343 18.098	0.597 0.692 0.691	0.661	0.854
Subjective Norm (SN)	0.893	SN1 SN2 SN3 SN4 SN5	3.99 3.86 3.88 3.76 3.74	0.879 0.860 0.848 0.864 0.895	0.726 0.797 0.829 0.818 0.791	- 20.616 19.367 17.011 19.793	0.527 0.626 0.687 0.670 0.625	0.629	0.894
Perceived Enjoyment (ENJ)	0.844	ENJ1 ENJ2 ENJ3 ENJ4	4.23 4.20 4.25 3.94	0.742 0.754 0.743 0.841	0.735 0.780 0.804 0.726	- 16.430 16.133 15.029	0.540 0.609 0.646 0.527	0.581	0.847
Technology Optimism (TO)	0.810	TO1 TO2 TO3 TO4	4.21 4.24 4.31 4.48	0.729 0.771 0.721 0.654	0.706 0.732 0.750 0.698	- 14.432 14.853 13.746	0.498 0.535 0.563 0.488	0.521	0.813
Perceived Trust (PT)	0.809	PT1 PT2 PT3 PT4	3.93 4.04 3.93 4.11	0.830 0.788 0.860 0.820	0.715 0.699 0.737 0.720	- 15.128 13.722 13.846	0.512 0.488 0.544 0.518	0.516	0.810

**Table 4:** Correlation matrix of the constructs.

	CR	AVE	MSV	ASV	SQ	FAM	TO	SN	ENJ	PT
SQ	0.850	0.587	0.595	0.435	0.766					
FAM	0.854	0.661	0.662	0.401	0.595 ***	0.813				
ENJ	0.847	0.581	0.618	0.453	0.704 ***	0.649 ***	0.763			
SN	0.894	0.629	0.576	0.423	0.759 ***	0.570 ***	0.705 ***	0.793		
TO	0.813	0.521	0.662	0.475	0.648 ***	0.814 ***	0.785 ***	0.614 ***	0.722	
PT	0.810	0.516	0.595	0.443	0.771 ***	0.615 ***	0.753 ***	0.742 ***	0.702 ***	0.718

Note: *** *p* < 0.01; MSV stands for the maximum shared variance; ASV stands for average shared variance.

**Table 5:** Goodness-of-fit test.

Category	Measure	Acceptable Values	Value
Absolute fit indices	Chi-square		479.9
	d.f.		238
	Chi-square/d.f.	1–5	2.016
	GFI	0.90 or above	0.922
	SRMR	0.08 or below	0.025
	RMSEA	0.05–0.08	0.046
Incremental fit indices	NFI	0.90 or above	0.928
	IF	0.90 or above	0.963
	TLI	0.90 or above	0.956
	CFI	0.90 or above	0.962

Note: GFI = goodness-of-fit index; SRMR = standardized root mean square residual; RMSEA = root mean square error of approximation; NFI = normed fit index; IFI = incremental fit index; TLI = Tucker-Lewis index; CFI = comparative fit index.

**Table 6:** Hypothesis testing.

	Path Direction	Standardized Coefficient	Standard Error	C.R. (t-Value)	Result
H1	SQ → SN	0.648 ***	0.060	10.820	Accepted
H2	SQ → PT	0.325 ***	0.065	4.199	Accepted
H3	SQ → ENJ	0.165 *	0.064	2.238	Accepted
H4	SQ → TO	0.196 ***	0.040	4.797	Accepted
H5	SQ → FAM	0.595 ***	0.046	10.651	Accepted
H6	FAM → SN	0.192 ***	0.064	3.672	Accepted
H7	FAM → ENJ	−0.068	0.092	−0.792	Rejected
H8	FAM → PT	0.012	0.088	0.140	Rejected
H9	FAM → TO	0.659 ***	0.058	10.155	Accepted
H10	SN → PT	0.233 **	0.061	3.201	Accepted
H11	SN → ENJ	0.282 ***	0.058	4.207	Accepted
H12	ENJ → PT	0.227 **	0.087	2.536	Accepted
H13	TO → ENJ	0.563 ***	0.122	5.540	Accepted
H14	TO → PT	0.159	0.136	1.366	Rejected

Note: * *p* < 0.1; ** *p* < 0.05; *** *p* < 0.01.

## Data Availability

The data presented in this study are available on request from the corresponding author.

## Appendix A

The following is the main questionnaire we used in this study. It consists of two parts, the demographic part and the motivation measurement part. For each demographic item, the options have been included in the brackets below. For each measurement question, five answers can be selected: “Strongly disagree”, “Disagree”, “Neutral”, “Agree” and “Strongly Agree”.

### Appendix A.1. Demographics

Age (15–20, 21–30, 31–40, 41+), Gender (Male, Female), Education Level (Some colleges, Undergraduate, Postgraduate), General Attitude (Acceptance in Home Motion-Sensing Game on a 5-point Likert Scale: Low to High; Future for Home Motion Sensing Game on a 5-point Likert Scale: Pessimistic to Optimistic).

### Appendix A.2. Measurement

System Quality (SQ)

- SQ1: Voice-enabled smart homes enable me to control many applications and devices.
- SQ2: Voice-enabled smart homes perform their functions quickly, efficiently, and precisely.
- SQ3: Voice-enabled smart homes and the output of information fully meet my needs.
- SQ4: Voice-enabled smart homes can protect my privacy and security.

Familiarity (FAM)

- FAM1: I am familiar with the concept and related knowledge about voice-enabled smart homes devices.
- FAM2: I am familiar with the brands, products, and services of voice-enabled smart homes devices.
- FAM3: I am familiar with how to operate voice-enabled smart homes devices.

Subjective Norm (SN)

- SN1: I will use voice-enabled smart homes devices if the media/government encourages to use them.
- SN2: I will use voice-enabled smart homes devices in my house if my family members and friends do so.
- SN3: I will use voice-enabled smart homes devices if people whose opinion I value recommend me that I so.
- SN4: I will use voice-enabled smart homes devices if people who influence my behavior recommend that I do so.
- SN5: I will use voice-enabled smart homes devices if people who are most important to me support me to do so.

Perceived Enjoyment (PE)

- ENJ1: Using voice-enabled smart homes would be fun.
- ENJ2: Using voice-enabled smart homes would be pleasurable.
- ENJ3: Using voice-enabled smart homes would be enjoyable.
- ENJ4: Using voice-enabled smart homes would make me excited.

Technology Optimism (TO)

- TO1: I feel that the newest technologies contribute to a better quality of life.
- TO2: I feel that the products and services that use the newest technologies are much more convenient to use.
- TO3: I feel confident that the newest technology-based systems will follow through with what I instruct them to do.
- TO4: I feel that the newest technologies can allow me to tailor things to fit my own needs.

Perceived Trust (PT)

- PT1: I consider voice-enabled Smart Homes to be trustworthy.
- PT2: I consider voice-enabled Smart Homes to be reliable.
- PT3: I consider voice-enabled Smart Homes to be controllable.
- PT4: I consider voice-enabled Smart Homes to be competent.
